# Numerical investigation of geomechanical responses of pillar during sequential stope-and-fill operations in squeezing rock

**DOI:** 10.1038/s41598-026-51689-3

**Published:** 2026-05-25

**Authors:** Ruofan Wang, Shichen Song, Lang Liu, Junjun Yang, Jiaxuan Wang, Yujie Zhu, Hao Cui

**Affiliations:** 1 College of Energy and Mining Engineering, Xi’an University of Science and Technology, Xi’an, 710054 China; 2 Key Laboratory of Western Mine Exploitation and Hazard Prevention Ministry of Education, Xi’an, 710054 China; 3 PowerChina Huadong Engineering Corporation Limited, Hangzhou, 311122 China; 4 Zhejiang Provincial Key Laboratory of Intelligent Prevention and Control for Safety of Trans-portation Infrastructure, Hangzhou, 311122 China

**Keywords:** Mechanical response, Time-dependency, Backfill, Pillar, Numerical model, Energy science and technology, Engineering, Solid Earth sciences

## Abstract

Mechanical performance of the intervening pillar critically influences extraction efficiency and operational safety in multiple stope-and-fill mining operations. However, the time-dependent interactions between backfill and creeping rocks and the resulting impact on pillar behavior remain poorly quantified, posing significant challenges in scheduling optimization, designs of pillar and backfill. This study employed FLAC3D to investigate the time-dependent geomechanical responses of pillar during sequential stope-and-fill operations, explicitly incorporating volumetric hardening behavior of consolidated backfill and creep behavior of rock mass. Results indicate that a short stope sequencing interval enables the consolidated backfill in the first stope to provide immediate lateral confinement to the pillar upon excavation of the second stope, improving stability. Conversely, extended intervals allow greater rock creep and enhance backfill densification and swelling pressure that eventually causes a beam-like bending of the pillar after adjacent excavation. It further leads to an asymmetric stress state characterized by tensile minimum principal stress *σ*₃ in the outer region (adjacent to the new stope), and high differential stress and elevated brittle shear ratio BSR in the inner part (against the consolidated backfill), marking the most critical state of pillar instability. An explicit dual-indicator framework based on *σ*₃ and BSR were proposed to capture this asymmetric mechanical response. Substantial parametric analyses were conducted to analyze the effects of stope geometry, pillar width, mine depth, backfill compression and swelling indexes, and rock viscosity on the results. Notably, pillar stability significantly recovered once the second stope was backfilled. These insights provide a mechanistic basis for optimizing the extraction strategy and the design of backfill and pillar in squeezing ground to minimize dilution and enhance operational safety and efficiency.

## Introduction

Backfill composed of tailings or waste rocks has become an integral part of underground mining to maintain ground stability and increase ore recovery rate with less dilution, which also promotes the environmentally friendly management of mine wastes^1–5^. The stabilizing effect of backfill on surrounding rock mass has been confirmed by in situ measurements, including reduced rock wall closure of backfilled stopes^6^ and decreased microseismic activities^7,8^. Therefore, a good understanding of the geomechanical responses of backfilled stopes is essential to assess the instability potential and optimize the extraction strategy, ground support, backfill and pillar design^9–11^. Significant research efforts have advanced the characterizations of stress development within single or sequentially backfilled stopes, as well as the stability of backfill itself during adjacent extractions^12–21^. Nevertheless, until now, very limited studies adequately account for the influence of backfill on the surrounding rock mass and ground stability. This critical gap between theoretical understanding and field evidence stems from the overlooking of long-term deformation of squeezing rocks and volumetric hardening behavior of backfill during consolidation.

**Fig. 1:**
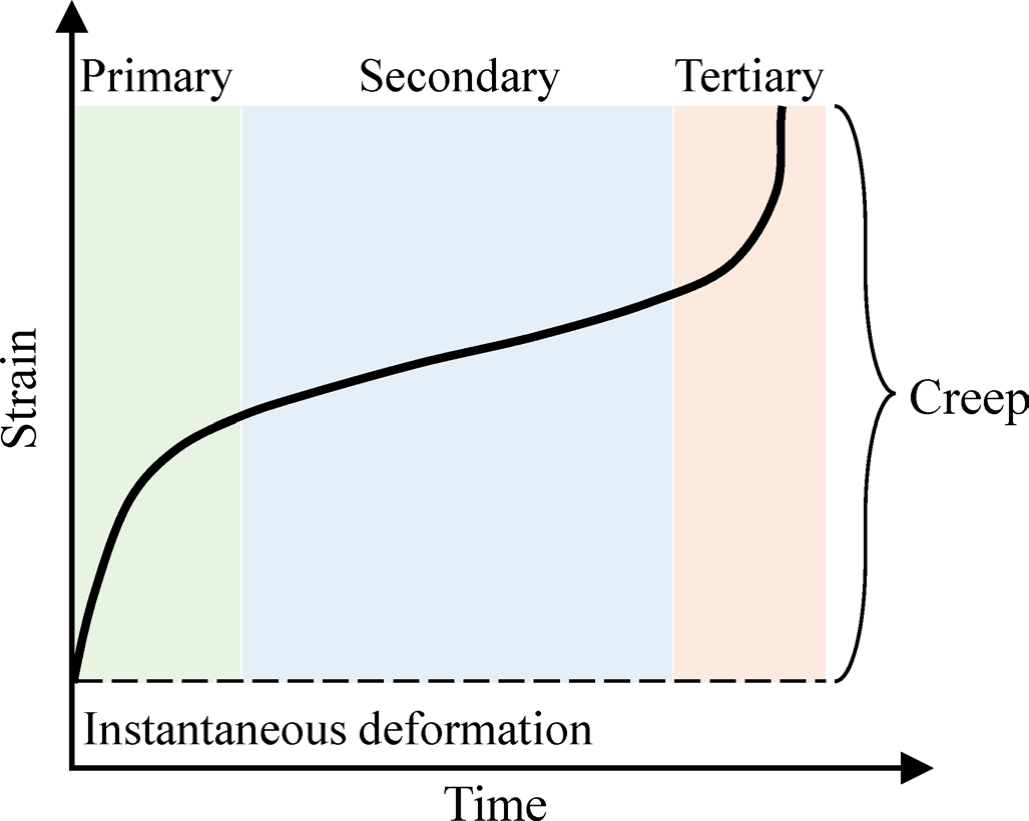
Schematic representation of rock creep strain.

When a rock is subjected to a constant stress condition (lower than its short-term strength), it can exhibit a time-dependent deformation, usually accompanied by some microseismic events that is known as creep behavior^22–24^. Figure 1 shows the schematic creep strain of rocks that typically consists of the primary creep stage with a decelerating creep strain rate; the secondary creep stage with an almost constant creep strain rate, and the tertiary creep stage with an accelerating creep strain rate leading to the delayed failure if the applied stress exceeds a threshold^25–28^. Most rocks are susceptible to creep behavior depending on the applied stress state^29–32^ and ambient thermal regime^33^. The creep behavior can result in significant squeezing condition of underground stope that has been identified in extensive underground mines even in those with hard surrounding rocks^34–39^.

In contrast, under the rock walls closure, the backfill placed in void stope can undergo consolidation with volumetric hardening^40–44^ that significantly affects its compressibility. The stress interactions between consolidated backfill and squeezing rock walls can thus impact the geomechanical behavior of the rocks around backfilled stopes. Recently, some exploratory studies have provided important insights into this aspect, which revealed a time-dependent stress redistribution mechanism and ground stability improvement around the backfilled stope in squeezing ground^45,46^. However, these studies focused solely on the geomechanical responses around a single vertical or inclined backfilled stope. Practically, backfills are applied in sequential stope-and-fill mining operations with an excavation-support sequence commonly called “leap frog”, wherein primary stopes are mined and backfilled while adjacent secondary stopes are temporarily left as intervening pillars^18,47–49^. This intervening pillar governs the integrity of the entire stope-and-fill operation, for example in sublevel or long-hole stoping methods, as its geomechanical performance directly impacts ore recovery efficiency and operational safety^14,50–52^. Nevertheless, the geomechanical behavior of the intervening pillar during sequential stope-and-fill operations particularly with consideration of stope sequencing interval remains inadequately addressed, posing significant challenges in mining scheduling optimization, designs of pillar and backfill.

To bridge this knowledge gap, numerical investigations were conducted using FLAC3D to examine the geomechanical responses of the pillar during sequential stope-and-fill mining in squeezing ground. The analysis explicitly incorporated time-dependent creep of rock mass using the Burger-Mohr model, consolidation behavior of backfill via the Soft Soil model, and the sequential excavation and backfilling of the second stope across a range of stope sequencing intervals. The numerical model was designed for sublevel or long-hole stoping methods that extract ore in a single blast and employ delayed backfill with primary and secondary stope sequencing strategy. The mechanism governing the geomechanical responses of intervening pillar was analyzed.Substantial parametric analyses were carried out to link key design variables including the stope sequencing interval, stoep geometry, pillar size, mine depth, compressibility of backfill and creep behavior of rocks to the time-dependent risk of instability in the intervening pillar.

## Modeling methods and programs

**Fig. 2:**
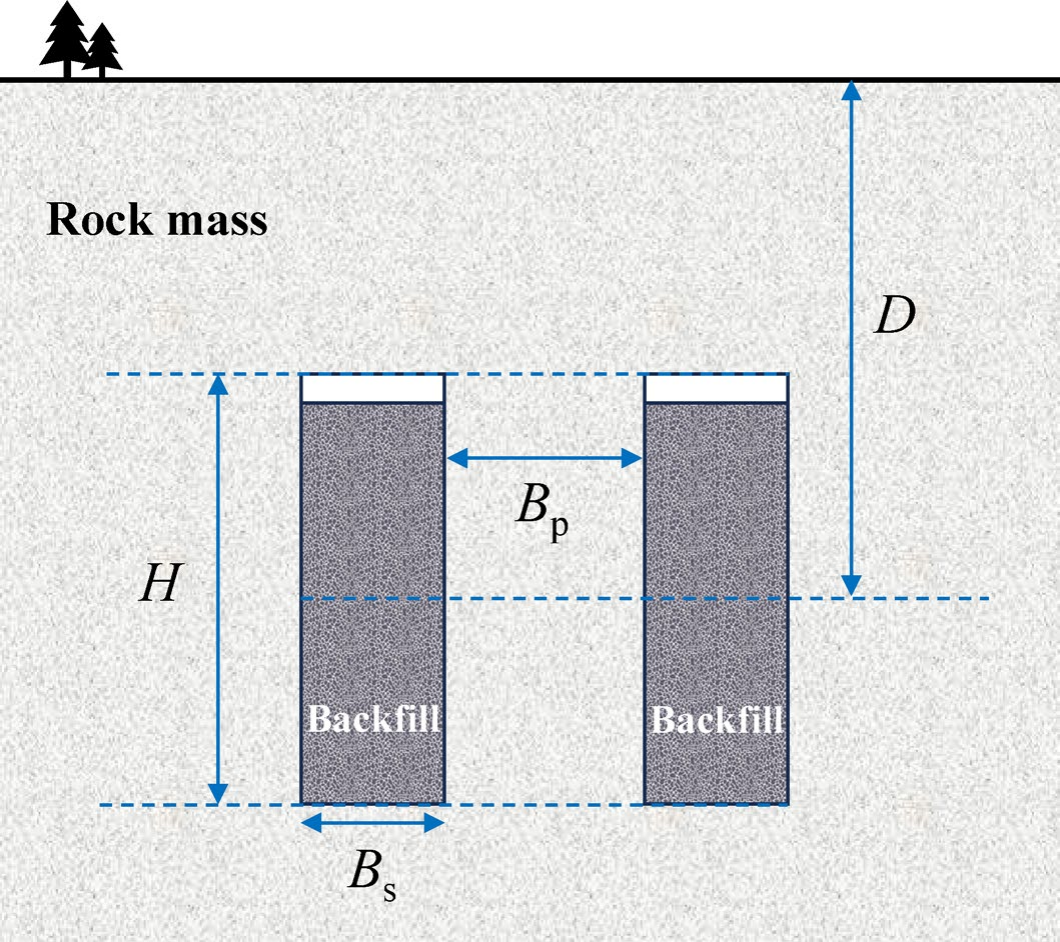
Conceptual model of two adjacent sequentially excavated and backfilled vertical stopes, separated by a pillar.

Figure 2 presents a conceptual model with two neighboring vertical stopes sequentially excavated and backfilled, and separated by a pillar. The backfilled stopes are located at a mine depth *D* (m) embedded within a rock mass characterized by long-term creep behavior. In the figure, *H* (m) represents the height of the stope, whereas *B*_s_ (m) and *B*_p_ (m) are the widths of the stope and the pillar, respectively. A filling gap with a height of *H*_g_ (m) remains beneath the stope roof which can result from drainage, desiccation and/or backfill consolidation. This problem will be treated by numerical modeling with FLAC3D.

**Fig. 3:**
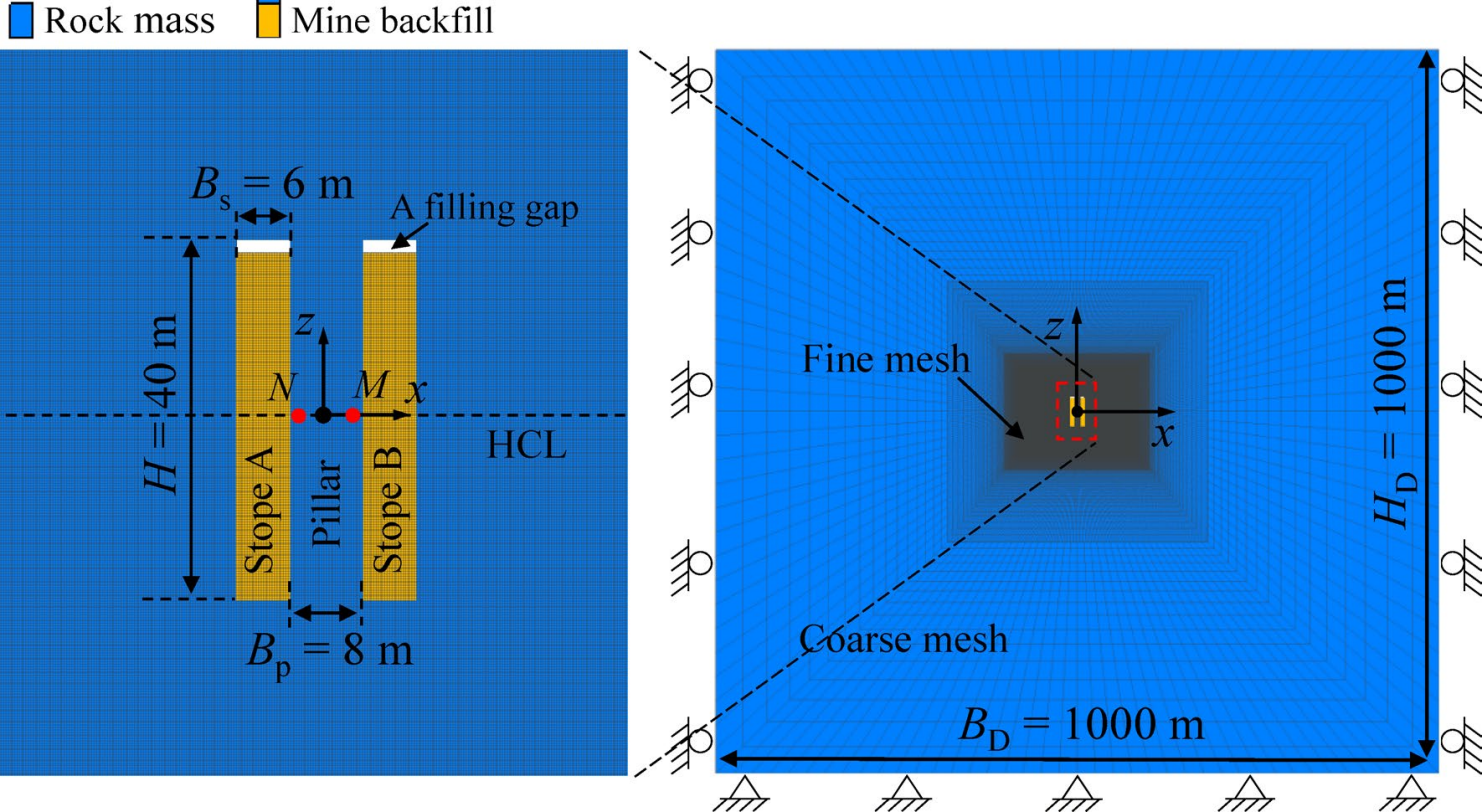
A plane strain numerical model of two vertical sequential backfilled stopes within a rock mass for a reference case, built with FLAC3D.

Figure 3 shows a two-dimensional plane strain numerical model, constructed using FLAC3D code^53^ of two adjacent backfilled stopes surrounded by a rock mass for a reference scenario. The stopes were extracted and backfilled in sequence and were defined as stopes A and B, respectively. The filling gap height *H*_g_ was set to 1.5 m while in practice, it can reach 3 to 5 m to accommodate the passage of vehicles and personnel^54^. The numerical model was developed with the primitive-based grid of radial tunnel in FLAC3D that incorporates brick-shaped elements and radially graded mesh. To ensure the reliability of the simulation, the height *H*_D_ (m) and the width *B*_D_ (m) of the numerical model were assigned a value of 1000 m based on the sensitivity analyses, thus avoiding influences from artificial boundary truncation on the results. The dimension along the out-of-plane (*y*-axis) was set to one unit (0.25 m) that is significantly smaller than that along the in-plane directions (*x*- and *z*-axes) to simulate the plane strain condition. The mine depth *D* (m) was measured form the ground surface to the coordinate origin (central) point. It is noteworthy that the value of *H*_D_ can change across different cases depending on the corresponding *D*. The fine brick-shaped meshes of 0.25 m were developed for backfilled stopes and immediate rocks, based on the sensitivity analyses. The far-field rock mass was discretized using coarse radially graded mesh with a mesh size that is integer multiple to the fine mesh. This designated meshing strategy was developed in a previous work^18^ and effectively represents the steep gradient in stress and displacement around the excavations while minimizing the mesh discontinuity and computational time.

In the numerical model, the gravity is along the negative direction of the z-axis. To establish an initial equilibrium state for the creep model^5,45,55–57^, a lateral earth pressure coefficient *K*_r_ = 1 was applied to determine the pre-mining horizontal stress based on the overburden condition. The displacement along the out-of-plane direction was prohibited and the lateral boundaries were set as roller boundaries to represent the plane strain condition. All displacements were fixed at the bottom to simulate the rigid bedrock, and the top surface was left as a free boundary where a surcharge was applied in some cases with large *D*. In Fig. 3, two symmetric points *N* and *M* as stresses monitoring locations were also marked along the horizontal central line (HCL) in the pillar. The two points were located close to the stopes A and B respectively and were positioned lightly inward (0.18 *B*_p_) from the outer pillar faces. These interior locations were chosen to avoid the local excavation and boundary relaxation effects at the immediate pillar surfaces.

The rock mass was considered obeying the Burgers-Mohr model^53,58,59^, that comprises a Burgers model and a Mohr-Coulomb (MC) model in series, characterized by the visco-elasto-plastic behavior. The Burgers model combines a Kelvin-Voigt element and a Maxwell element, and is responsible for capturing the creep behavior. Once the stress state exceeds the threshold stipulated by the MC criterion, the plastic yield will be simulated through the activation of the MC model. The applicability of the Burgers-Mohr model has been extensively validated for representing the time-dependent creep behavior of various rock types such as sandstone, rock salt, schist and limestone, through comparisons with compression creep test results^5,60–62^, despite different creep mechanisms for hard and typical soft rocks^23,28,63^. Owing to its adaptable capability, the Burgers-Mohr model is prevalently used in describing creep behavior of rocks^7,45,64,65^. The viscoelastic creep response of rock mass is characterized by a bulk modulus *K*_R_ (GPa), a shear modulus *G*_K_ (GPa) and a viscosity coefficient *η*_K_ (Pa⋅s) of the Kelvin-Voigt element, as well as a shear modulus *G*_M_ (GPa) and a viscosity coefficient *η*_M_ (Pa⋅s) of the Maxwell element. Moreover, the rock mass is defined by a unit weight *γ*_R_ (kN/m^3^), a cohesion *c*_R_ (MPa), an internal friction angle *ϕ*_R_ (°), a dilation angle *ψ*_R_ (°), and a tensile strength *T*_R_ (MPa). It is worth noting that *G*_M_ and *K*_R_ of the rock mass can be derived from its Young’s modulus *E*_R_ (GPa) and Poisson’s ratio *ν*_R_ via Hooke’s law.

Table 1 gives the mechanical properties of rock mass used in the reference case, with values adopted within typical ranges suggested in the literature^5,60,66^.

**Table 1:** Mechanical properties of rock mass adopted in a reference scenario.

*γ*_R_ (kN/m^3^)	*E*_R_ (GPa)	*ν*_R_	*c*_R_ (MPa)	*ϕ*_R_ (°)	*ψ*_R_ (°)
27	40	0.25	8	40	0
*T*_R_ (MPa)	*K*_R_ (GPa)	*G*_K_ (GPa)	*η*_K_ (Pa·s)	*G*_M_ (GPa)	*η*_M_ (Pa·s)
0.9	26.7	3	3 × 10^14^	16	1 × 10^15^

The volumetric hardening behavior of backfill plays a crucial role in analyzing fill-rock interactions under the constraint from rock walls closure^43,45,46^. Accordingly, the dry backfill in the numerical model is simulated using the Soft Soil model which is in Cam-Clay type comprising a MC criterion and elliptical volumetric yield surface^67^. The Soft Soil model represents the consolidation behavior of backfill through a logarithmic relationship between the mean stress *p* (MPa) and the volumetric strain *ε*_v_. The validity of this model in capturing the volumetric hardening and stress-dependent stiffness of lowly cemented backfill has been established by numerically reproducing one-dimensional consolidation and consolidated-drained triaxial compression test results, as detailed in prior works^41–43^. One should note that for mining backfill represented by the Soft Soil model, the compressibility during volumetric hardening is governed by two key parameters: the modified compression index λ^*^ and the modified swelling index κ^*^. They can be derived from the conventional compression index *C*_c_ and swelling index *C*_s_ of the backfill per the following equations^53^:

1 $$\:{\lambda\:}^{*}=\frac{{C}_{\mathrm{c}}}{2.3(1\:+\:e)}$$

2 $$\:{\kappa\:}^{*}\approx\:\frac{\left(1+2{K}_{0}\right)(1-\nu\:)}{(1+\nu\:)}\frac{{C}_{\mathrm{s}}}{2.3(1+e)}$$

where *ν* is the Poisson’s ratio; *K*_0_ denotes the at-rest earth pressure coefficient defined based on the internal friction angle *ϕ* (°) as (1 − sin*φ*); *e* is the void ratio.

Additionally, the constitutive response of backfill is described by a unit weight *γ* (kN/m^3^), a cohesion *c* (MPa), a dilation angle *ψ* (°), a tensile strength *T* (kPa), a preconsolidation pressure *p*_c_ (kPa), and an initial void ratio *e*_ini_. Table 2 summarizes the mechanical properties of the backfill employed in a reference case considering test results reported in the literatures^41,68–71^. In this study, *ν* of backfill is related to its *ϕ* through the expression *ν* = (1 − sin*φ*)/(2 − sin*φ*) to ensure a consistent *K*_0_^15,18,45,72,73^.

**Table 2:** Mechanical properties of backfill applied in a reference scenario.

*γ* (kN/m^3^)	*λ* ^*^	*κ* ^*^	*c* (MPa)	*ϕ* (°)	*ν*
18	2.41 × 10^− 2^	2.43 × 10^− 3^	0	30	0.33
*T* (kPa)	*ψ* (°)	*K*_0_	*p*_c_ (kPa)	*e*_ini_	
0	0	0.5	10	0.96	

In the numerical model, the timestep in a creep calculation defines a physical time span which affects the geomechanical response and differs from the conventional static simulation. Therefore, the timestep should be adjusted to maintain the numerical model in a quasi-static mechanical equilibrium state^53^. The necessary model parameters include: the initial timestep *t*_i_ (s), the minimum timestep *t*_min_ (s), the maximum timestep *t*_max_ (s), the latency, the upper boundary *ufb* and lower boundary *lfb* of the unbalanced force ratio (UFR). The timestep was increased by a factor of 1.02 as UFR was lower than *lfb*. Conversely, it reduced by applying a multiplier 0.98 once UFR exceeds *ufb*. Notably, the limit of *t*_max_ must be restricted to ensure the time-dependent stress variation within each timestep remains smaller than the strain-dependent stress changes. This limit can be determined using the equation suggested by Itasca^53^:

3 $$\:{t}_{\mathrm{m}\mathrm{a}\mathrm{x}}=\mathrm{m}\mathrm{i}\mathrm{n}\left(\frac{{\eta\:}_{\mathrm{K}}}{{G}_{\mathrm{K}}},\frac{{\eta\:}_{\mathrm{M}}}{{G}_{\mathrm{M}}}\right)$$

In the numerical models, *t*_max_ was taken as 1000 s; *t*_min_ was taken as 1 × 10^− 3^ s; *t*_i_ was defined as 1 s; latency was set to 100 steps; *ufb* was 1 × 10^− 5^ and *lfb* was 8 × 10^− 6^, based on a sensitivity analysis.

**Fig. 4:**
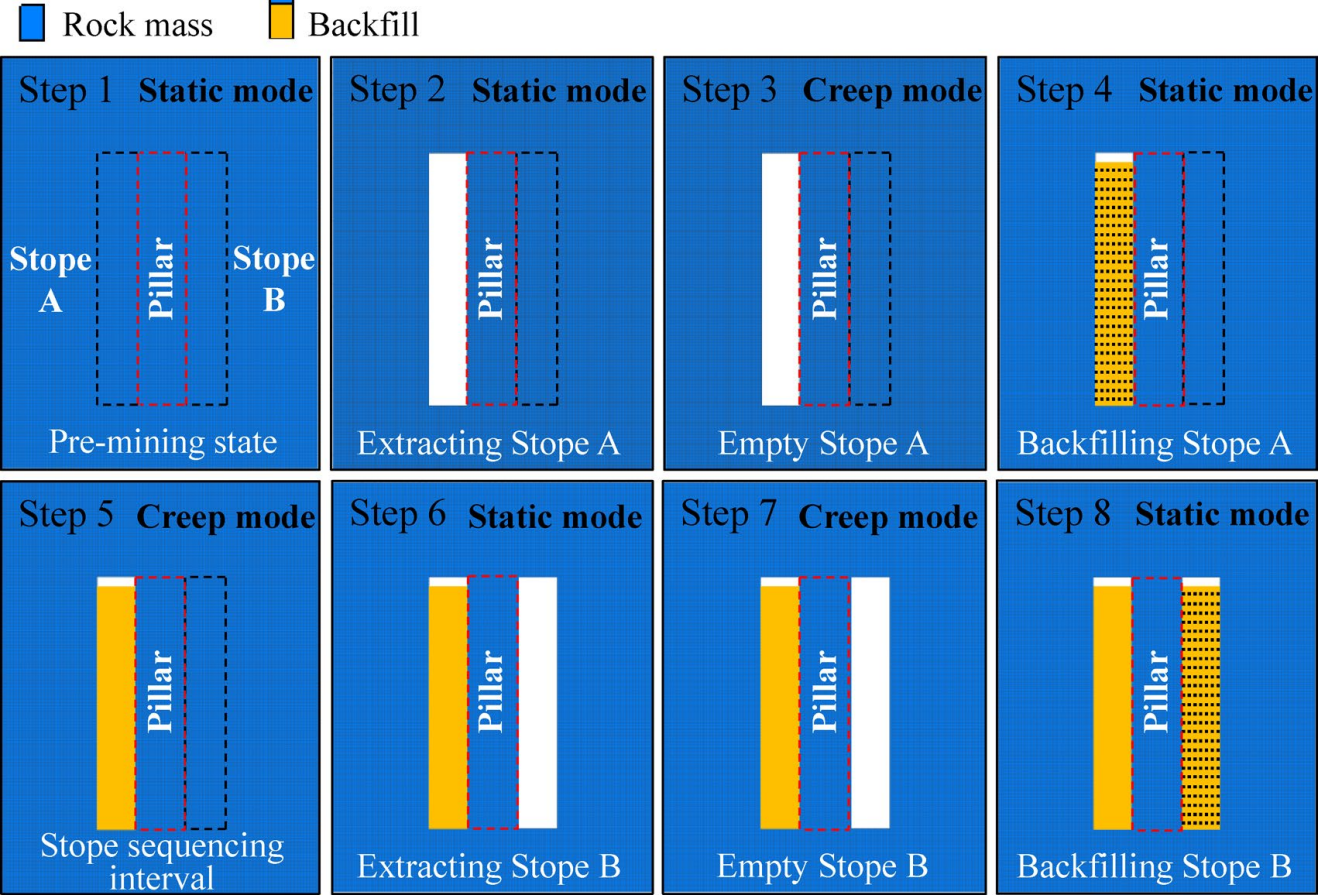
Numerical simulation steps for sequential stope-and-fill operations within squeezing rock condition.

The influence of sequential stope-and-fill operations on inter-stope pillar stability was analyzed through a series of simulation steps as illustrated in Fig. 4, alternating between static and creep calculation modes. The numerical model was initially computed to reach the pre-mining (in-situ) stress state under static condition. Subsequently, Stope A was extracted in the same static analysis framework. Following the extraction, the immediate backfilling of the stope was delayed due to the operational requirements, including mucking, ground inspection, ventilation, and fill material scheduling. Therefore, in Step 3, an empty stope exposure time *t*_e_ representing the typical delay between excavation and backfilling was simulated for the void Stope A using the creep mode. Stope A was then backfilled in Step 4 in layers with 2 m thick per layer with static computational mode. The equilibrium state was obtained as each layer of backfill was placed. One should note that this layer-by-layer simulation is a critical technique to realistically represent the site condition where placed fill material remains in a mechanical equilibrium state^12,18^. In Step 5, the stope sequencing interval time *t*_s_ (d) was simulated by reactivating the creep analysis mode, which represents an elapsed time between the completion of Stope A and the start of extraction of adjacent Stope B. During *t*_s_, the backfill can undergo curing and preparation of the adjacent stope are made. Following this, the excavation, exposure, and layerly backfilling were simulated again for Stope B in Steps 6–8 employing aforementioned static and creep calculation modes, respectively. The complete numerical simulation steps are further summarized in Table 3. In the reference case, *t*_e_ was 7 d and *t*_s_ was taken as 50 d.

**Table 3:** Descriptions of the sequential steps in the numerical simulations.

Simulation steps	Descriptions
Step 1	Obtain the initial state before any excavation. (Static mode)
Step 2	Excavate the entire Stope A in a single step, and reach the equilibrium state. (Static mode)
Step 3	Simulate an exposure time *t*_e_ for the empty Stope A (Creep mode)
Step 4	Layerly backfilling Stope A. Ensure an equilibrium state after the placement of each layer. (Static mode)
Step 5	Simulate a stope sequencing interval time *t*_s_, during which the rock mass deforms time-dependently. (Creep mode)
Step 6	Excavate the entire Stope B in one step, and reach the equilibrium state. (Static mode)
Step 7	Simulate an exposure time *t*_e_ for the empty Stope B. (Creep mode)
Step 8	Layerly backfilling Stope B. Ensure mechanical equilibrium after the placement of each layer. (Static mode)

In the numerical model, the relative deformations between zones remained significantly smaller than the element dimensions, despite the long-term creep deformation of the rock mass. Therefore, both the small strain and the large strain computational modes are applicable for the simulations, as discussed by Wang et al.^45^. The small strain mode was used in this study. Table 4 provides the numerical modeling program of all cases conducted in this research. The influences of key factors on the geomechanical response of squeezing rocks around sequential stope-and-fill operation system were studied by varying one parameter at a time (denoted as VAR in the table) while keeping the others constant. Case 1 serves as the reference case in this work.

**Table 4:** Numerical modeling program.

Case	*t*_s_ (d)	Geometry			*D* (m)	Backfill		Rock mass
		*H* (m)	*B*_s_ (m)	*B*_p_ (m)		λ^*^ (× 10^− 2^)	κ^*^ (× 10^− 3^)	*η*_M_ (× 10^15^ Pa·s)
1	VAR	40	6	8	500	2.41	2.43	1
2	50	VAR	6	8	500	2.41	2.43	1
3	50	40	VAR	8	500	2.41	2.43	1
4	50	40	6	VAR	500	2.41	2.43	1
5	50	40	6	8	VAR	2.41	2.43	1
6	50	40	6	8	500	VAR	2.43	1
7	50	40	6	8	500	2.41	VAR	1
8	50	40	6	8	500	2.41	2.43	VAR

## Geomechanical response of pillar in sequential stop-and-fill operations

### Single stope

**Fig. 5:**
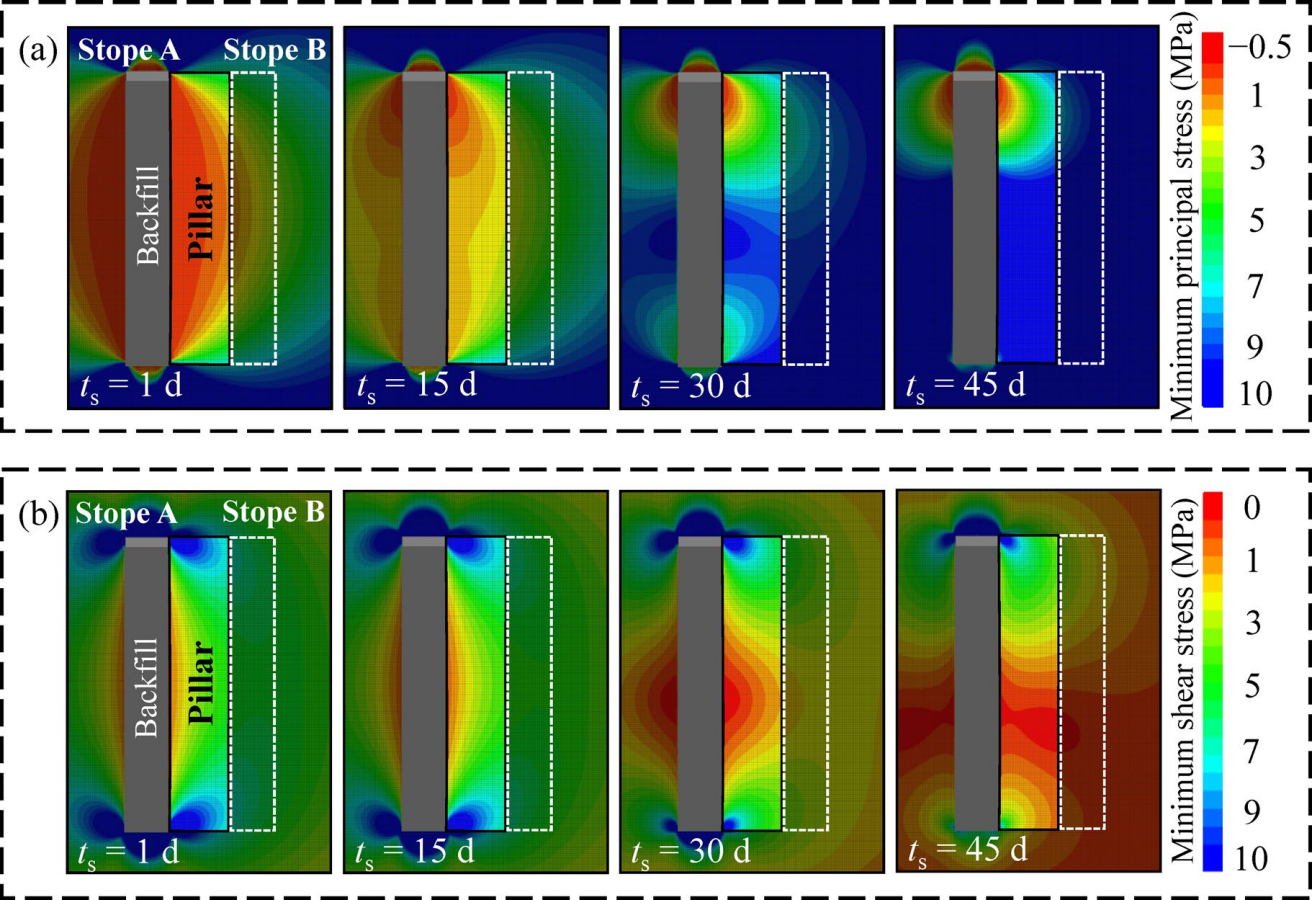
Contour-filled plots of (**a**) the minimum principal stress and (**b**) the maximum shear stress around the backfilled Stope A before excavation of Stope B for varying *t*_s_ in the reference case with emphasis on the intervening pillar.

Figure 5 presents the spatial distributions of the minimum principal stress *σ*_3_ and the maximum shear stress (*σ*_1_ − *σ*_3_)/2 (*σ*_1_ denotes the maximum principal stress) around the first backfilled stope (Stope A) prior to excavation of the second stope (Stope B), for different stope sequencing interval times *t*_s_ in the reference case. The time-dependent stress evolution within the intervening pillar is highlighted in bright colors. The compression is in positive throughout this study. Numerical results suggest that during the prolonged *t*_s_, *σ*_3_ around the single backfilled stope recovers from a low magnitude to the far field in-situ stress level of over 10 MPa. Concurrently, the maximum shear stress decreases obviously as *t*_s_ increases. The increased *σ*_3_ and the reduced differential stress collectively suggest that squeezing rocks around an isolated backfilled stope gain a time-dependent stability enhancement. These findings are consistent with the literatures on time-dependent stress redistribution around a single backfilled stope^45,46^.

**Fig. 6:**
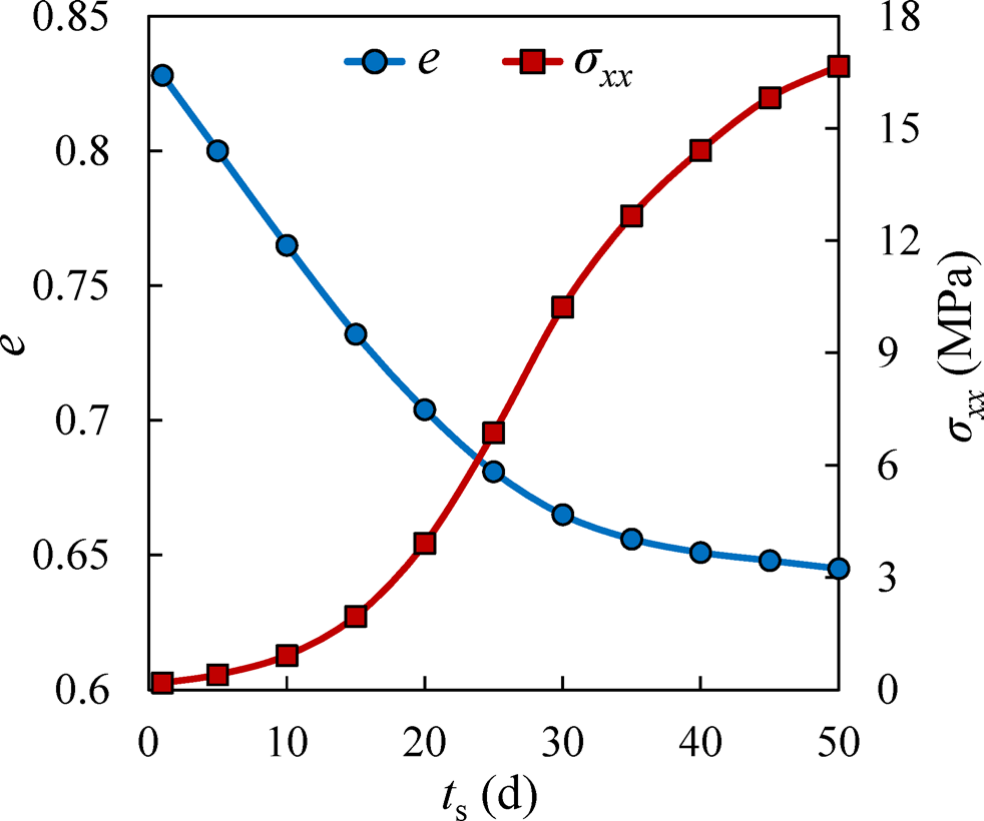
Evolutions of *e* and *σ*_*xx*_ at the center point of the backfilled Stope A as functions of *t*_s_ before Stope B extraction in the reference case.

The time-dependent improvement on ground stability around a single backfilled stope is further clarified by the evolutions of void ratio *e* and horizontal stress *σ*_*xx*_ at the center point of the backfilled Stope A as functions of *t*_s_ as depicted in Fig. 6. Numerical results reveal that the backfill in Stope A undergoes progressive consolidation as *t*_s_ increases, resulting in a denser and stiffer state with a reduced *e*. The consolidated backfill sustains greater compressive stresses induced by creep deformation of the surrounding rock walls, as evidenced by the rising *σ*_*xx*_ which delivers enhanced passive support on peripheral rocks^43,45,46^. These geomechanical responses confirm that the stress distribution and ground stability around an isolated backfilled stope evolve temporally, and is consistent with those shown in Fig. 5.

### Two adjacent stopes

**Fig. 7:**
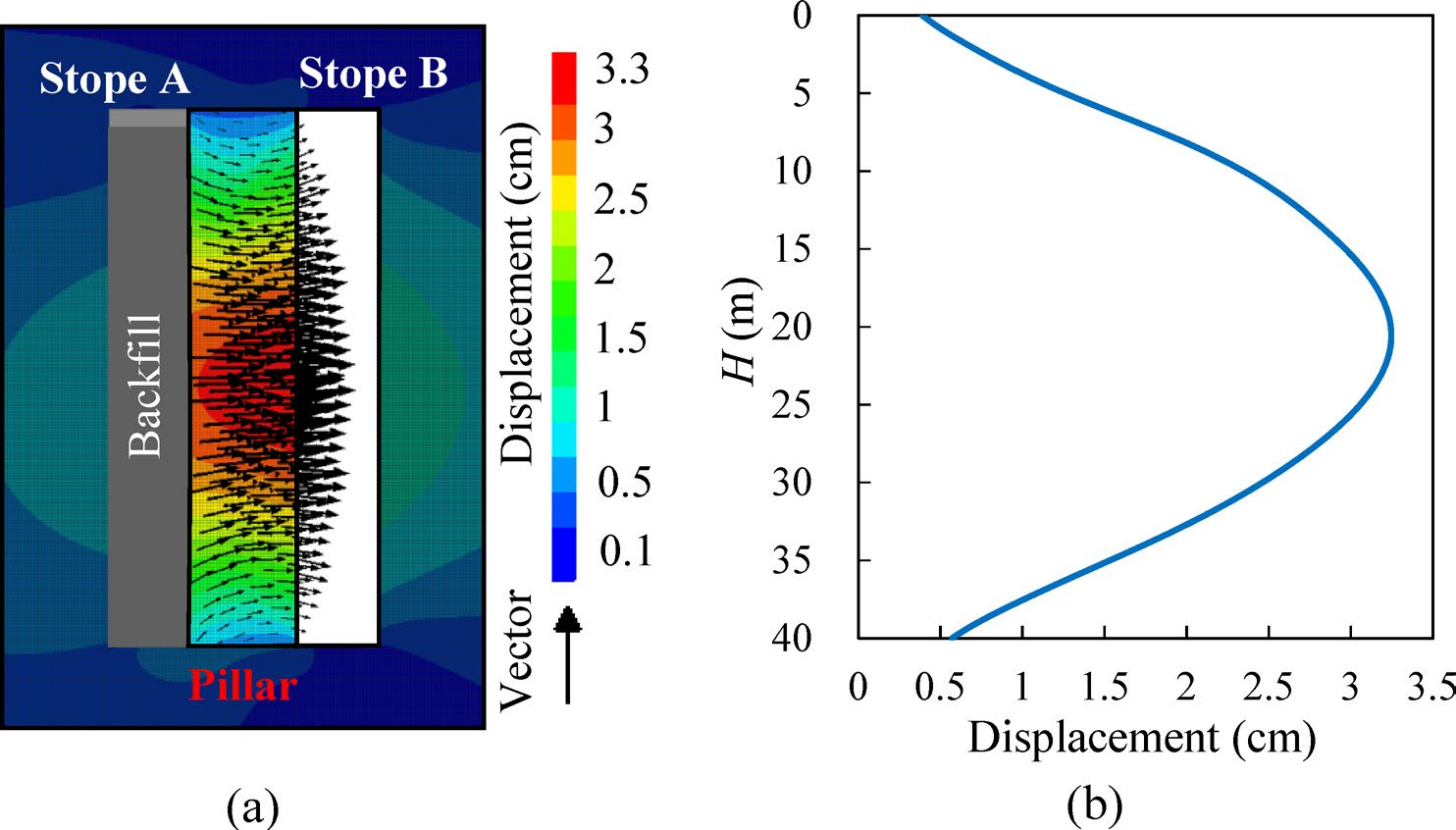
(**a**) Displacement distribution with corresponding vectors and (**b**) total displacement along the VCL of the pillar upon the extraction of the second stope after a *t*_s_ = 50 d in sequential stope-and-fill operations.

Despite the time-dependent improvement on the ground stability around a single backfilled stope, the geomechanical responses of intervening pillar during sequential stope-and-fill operations can be quite different. Basically, the pillar can experience a critical loss of stability during Step 6 when the second stope is excavated before subsequent backfilling in Step 8. Step 6 thus represents the most unfavorable condition for pillar stability that requires detailed evaluation. Figure 7a shows the displacement field and corresponding vectors of the pillar after excavation of Stope B, following a stope sequencing interval *t*_s_ = 50 d. The pillar exhibits a distinct beam-like bending deformation characterized by pronounced lateral displacement toward Stope B around its mid-height while displacements at the top and bottom remain minimal. This pattern is further confirmed by Fig. 7b, which plots the total displacement along the vertical central line (VCL) of the pillar and shows a peak value of 3.3 cm at the center. This behavior arises because creep of the surrounding rock during the sequencing interval compresses the backfill in Stope A, leading to high consolidation. Upon excavation of Stope B, the confined backfill partially unloads and exerts a sustained lateral swelling pressure on the inner face of the pillar. With this one-sided horizontal load applied over its height and the pillar top and bottom ends restrained by the surrounding rocks, the pillar deforms in a manner analogous to a vertically fixed beam under lateral loading, resulting in the observed beam-like bending response. Notably, this unique displacement pattern highly depends on *t*_s_ and consequently affects the stress distribution and overall stability of the pillar.

**Fig. 8:**
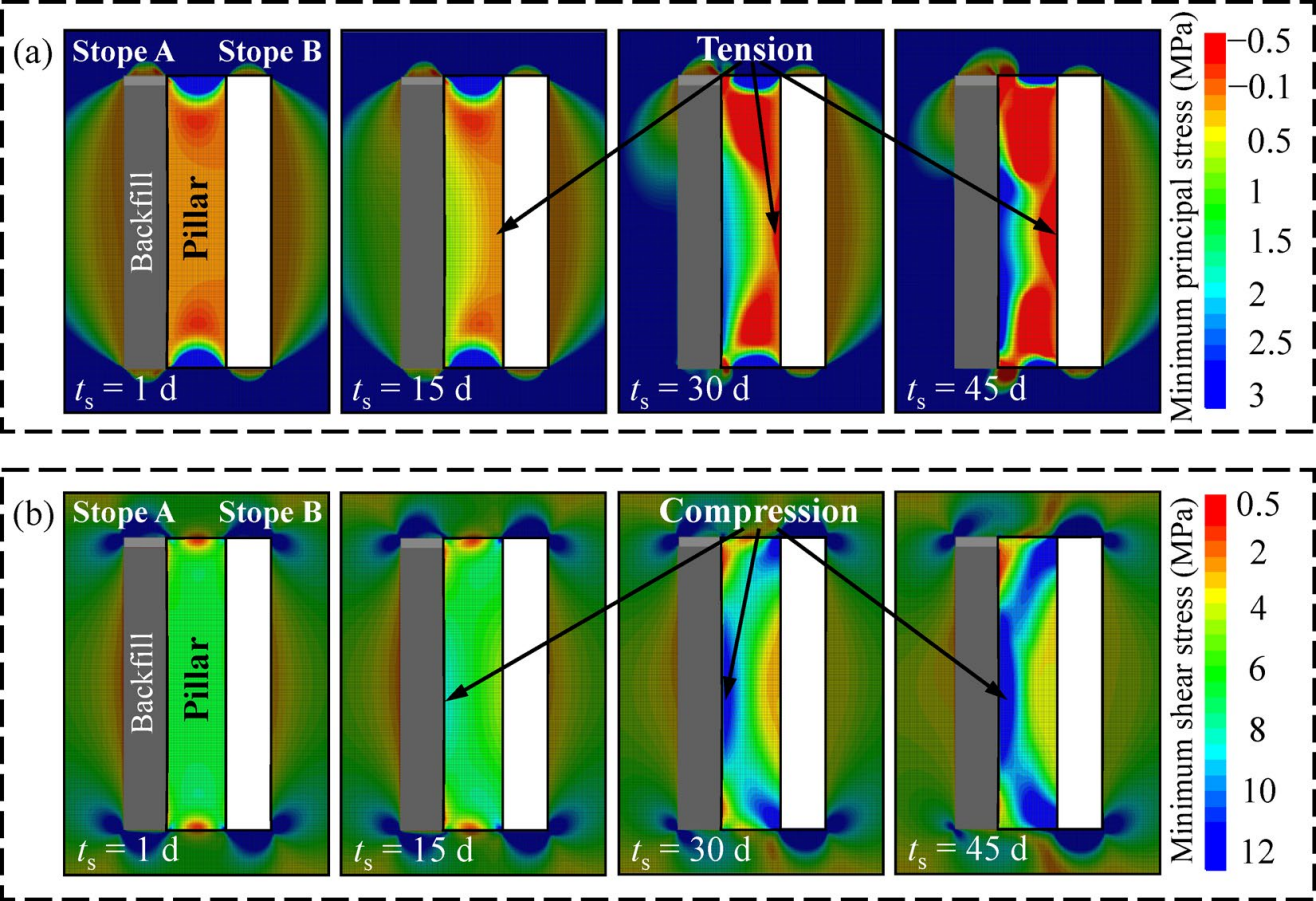
Contour-filled plot of (**a**) the minimum principal stress and (**b**) the maximum shear stress around two adjacent stope-and-fill operations upon the excavation of Stope B for varying *t*_s_ in the reference case, with emphasis on the pillar.

Figure 8 shows the distributions of *σ*_3_ and (*σ*_1_ − *σ*_3_)/2 around two adjacent stope-and-fill operations immediately after excavation of Stope B (Step 6) for varied *t*_s_ in the reference case, with focus on the intervening pillar. The beam-like bending displacement pattern results in a pronounced asymmetry in stress state within the pillar. The inner side experiences increased differential stress resulted from the swelling pressure from consolidated backfill and the bending effect. The outer region (adjacent to Stope B) demonstrates tensions reflected by reduced *σ*_3_ and lower differential stress. The asymmetrical stress condition becomes apparent as *t*_s_ is larger than 15 d, and intensifies as *t*_s_ increases. The asymmetrical stress states can potentially lead to a hybrid failure mode of the pillar that is tensile extension in the outer region and high-deviatoric brittle damage in the inner portion.

To characterize the time-dependent stability of the pillar in this asymmetrical failure mode, *σ*_3_ at a point *M* and the brittle shear ratio (BSR) at a point *N* (Fig. 3) were employed as the stability indicator. *σ*_3_ reflects the confinement effectiveness and the presence of tensile stress^45,46^. The peripheral rocks with low *σ*_3_ or tensile stress are typically potential for sloughing and strain bursts. Moreover, BSR is an instability indicator commonly used to analyze the brittle compressive failure of rocks^74–76^. It is defined as a ratio of the differential stress to the uniaxial compressive strength (UCS) of an intact rock, as given in Eq. (4). The seismicity of rocks induced by high differential stress can occur as BSR exceeds 0.3–0.4 while the brittle shear failure was observed for BSR larger than 0.7^74^.

4 $$\:\mathrm{B}\mathrm{S}\mathrm{R}=\frac{\left({\sigma\:}_{1}-{\sigma\:}_{3}\right)}{\mathrm{U}\mathrm{C}\mathrm{S}}$$

Different stability indicators on each side of the pillar were employed because they correspond to the asymmetrical failure mechanism as demonstrated in Fig. 8. *σ*_3_ in the outer region directly tracks tensile risk where fractures initiate, whereas BSR in the inner portion quantifies proximity to brittle compressive failure under confinement. The evaluation of pillar stability should combine the two stability indicators. Nevertheless, tensile conditions in the outer part are treated as the primary control criterion since spalling in this region directly leads to hanging-wall collapse, resulting in safety hazards and ore dilution in the production stope.

**Fig. 9:**
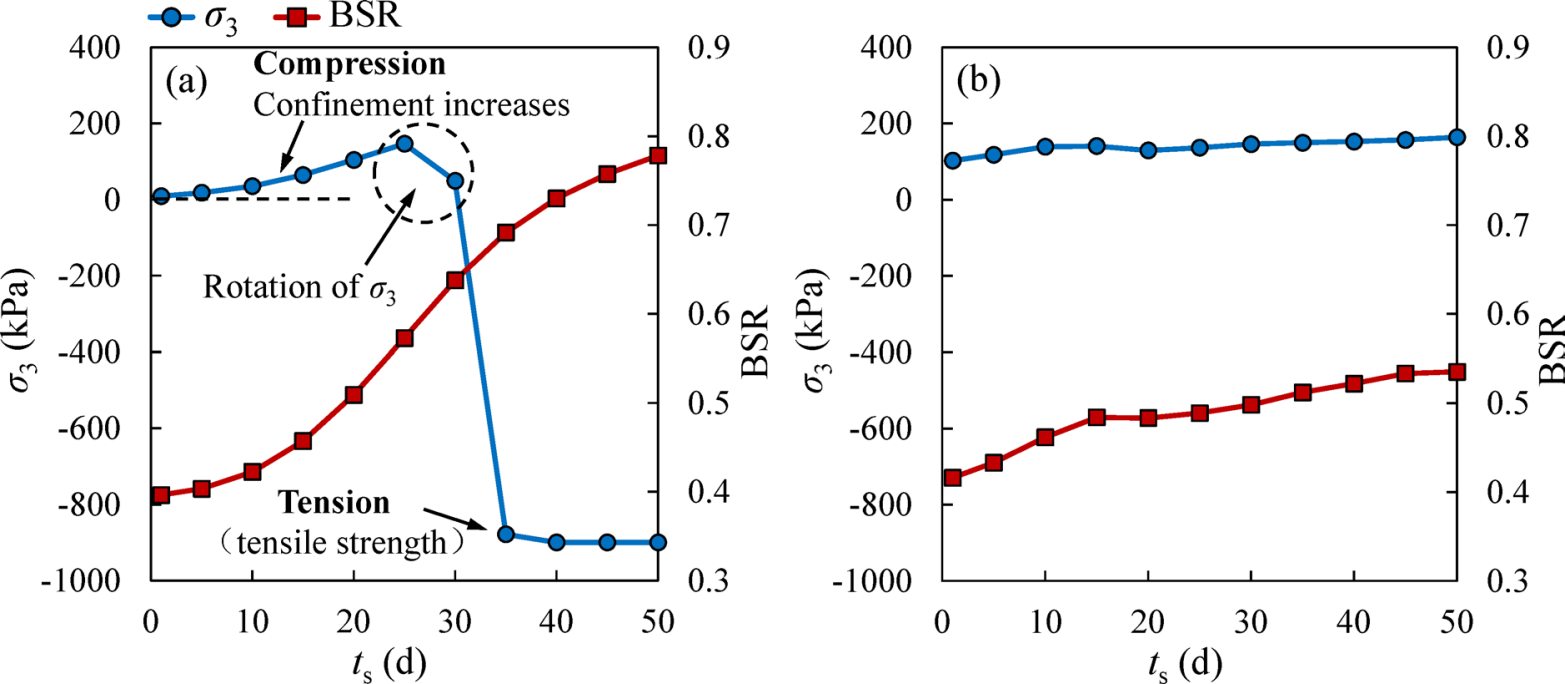
Evolutions of *σ*_3_ at the point *M* and BSR at the point *N* as functions of *t*_s_ after (**a**) the extraction (Step 6) and (**b**) the backfilling (Step 8) of Stope B in the reference case.

Figure 9 draws the evolutions of *σ*_3_ at the point *M* and BSR at the point *N* as functions of *t*_s_ upon extraction (Step 6) and after backfilling (Step 8) of stope B in the reference case. During the excavation of Stope B, *σ*_3_ in the outer region of the pillar increases as *t*_s_ increases from 1 to 25 d that indicates the improvements on the confinement effectiveness and the stability of the pillar. This is followed by a sharp decline in *σ*_3_ which becomes tensile with the negative value and eventually reaches the tensile strength 900 kPa of rocks as the stope sequencing interval continues increasing. It can be explained as longer *t*_s_ results in greater consolidation of the backfill in Stope A, leading to higher lateral confinement on the pillar when the mining cycle interval is not very large. Conversely, as *t*_s_ exceeds a threshold, the highly compressed backfill exerts substantial swelling pressure upon excavation of Stope B, inducing prominent bending pattern of the pillar and driving the outer part of the pillar into tension. This transition is accompanied by a reorientation of *σ*_3_ in the outer region of pillar, which shifts from horizontal compression to vertical tension as the bending-induced tensile stress along the pillar height becomes the new direction for the minimum principal stress. Concurrently, the BSR in the inner portion of the pillar increases monotonically with *t*_s_ and surpasses 0.7 when *t*_s_ is larger than 35 d, as a result of the bending deformation caused compression on the inner side. In this case, the stope sequencing interval time should be optimized to be less than 25 d based on two indicators to maintain pillar stability.

After Stope B backfilling in Step 8, Fig. 9b shows that stress state in the pillar undergoes significant improvement. The outer region of pillar remains compression regardless of the stope sequencing interval time, as the backfill in Stope B provides the lateral confinement. Meanwhile, BSR in the inner region of pillar still increases with the increase of *t*_s_, but its magnitudes substantially reduce compared with those during the Stope B excavation. This attenuation is attributed to that creep deformation of the pillar during the empty time of Stope B dissipates some of the bending-induced compression in the inner region of the pillar. Consequently, backfilling Stope B enhances overall pillar stability. Similar behavior has been observed across other cases.

## Parametric analyses

Numerical results indicate that the most critical condition for the intervening pillar occurs immediately upon excavation of Stope B while Stope A is already backfilled. This scenario governs pillar stability and requires detailed evaluation. In the following, the effect of different parameters on the geomechanical response of intervening pillar will be investigated in terms of its asymmetrical stability indicators *σ*_3_ and BSR upon the extraction of Stope B. The post-backfilling states of Stope B exhibit again improved stability and are not revisited in subsequent analyses.

### Stope geometry

**Fig. 10:**
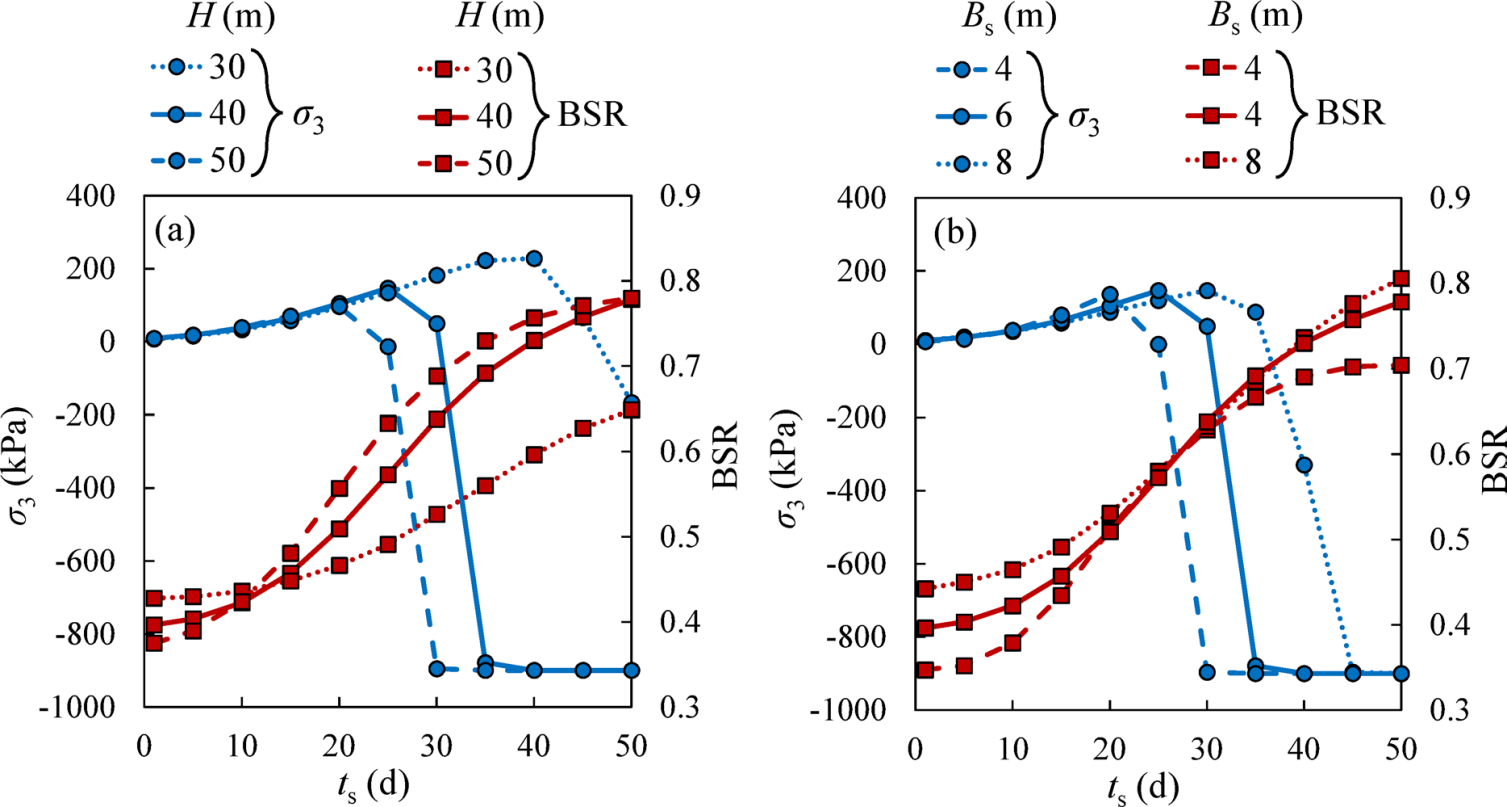
Variations of *σ*_3_ at the point *M* and BSR at the point *N* as functions of *t*_s_ after the extraction of Stope B for different (**a**) heights *H* and (**b**) widths *B*_s_ of stopes in Cases 2 and 3.

Figure 10 draws the variations of stability indicators *σ*_3_ at the point *M* and BSR at the point *N* as functions of stope sequencing interval time *t*_s_ immediately after the extraction of Stope B for different heights *H* and widths *B*_s_ of stopes in Cases 2 and 3. When *t*_s_ is small, results indicate that *σ*_3_ in the outer region of intervening pillar is almost independent of *H*, but it slightly increases with the decrease in *B*_s_. For small *t*_s_, BSR in the inner portion slightly reduces with the increase of *H*, whereas BSR moderately elevates as *B*_s_ increases. This is because for an excavation with a large *H* (high aspect ratio), the stress arch on the stope top efficiently redistributes the overburden to surrounding rock walls, partially unloading the vertical (maximum principal) stress in the intervening pillar. By contrast, a larger span (*B*_s_) of the excavation results in higher vertical loading and BSR in the pillar.

However, as *t*_s_ continuous increasing, a higher pillar exhibits the tensile condition at shorter earlier mining sequencing interval, with a steeper reduction rate for *σ*_3_ as well as a higher increasing rate for BSR. It can be understood as a higher pillar exhibits more pronounced transverse bending response and is thus more vulnerable. Moreover, the pillar adjacent to stopes with smaller *B*_s_ demonstrate tensions at earlier *t*_s_ with a higher reduction rate of *σ*_3_, but the overall magnitude of BSR is relatively lower than the case with larger *B*_s_. This is explained as the backfill in the narrow stope generates higher compressive strain and stress under rock walls creep deformation, which leads to significant swelling pressure and bending for the pillar after extracting Stope B.

In practice, the primary stopes must be filled with high cement additive to maintain backfill stable upon side exposure while the secondary stope (i.e., intervening pillar) can be filled with lowly cemented (or uncemented) backfill. Therefore, narrow primary stopes are usually preferred. Results in Fig. 10 suggest that sequential stope-and-fill operations with narrow primary stopes should explicitly shorten mining cycles, to mitigate the tensile spalling potential for intervening pillar. Meanwhile, more cautions are needed for the brittle shear failure of pillar neighboring large-span stopes.

### Pillar width

**Fig. 11:**
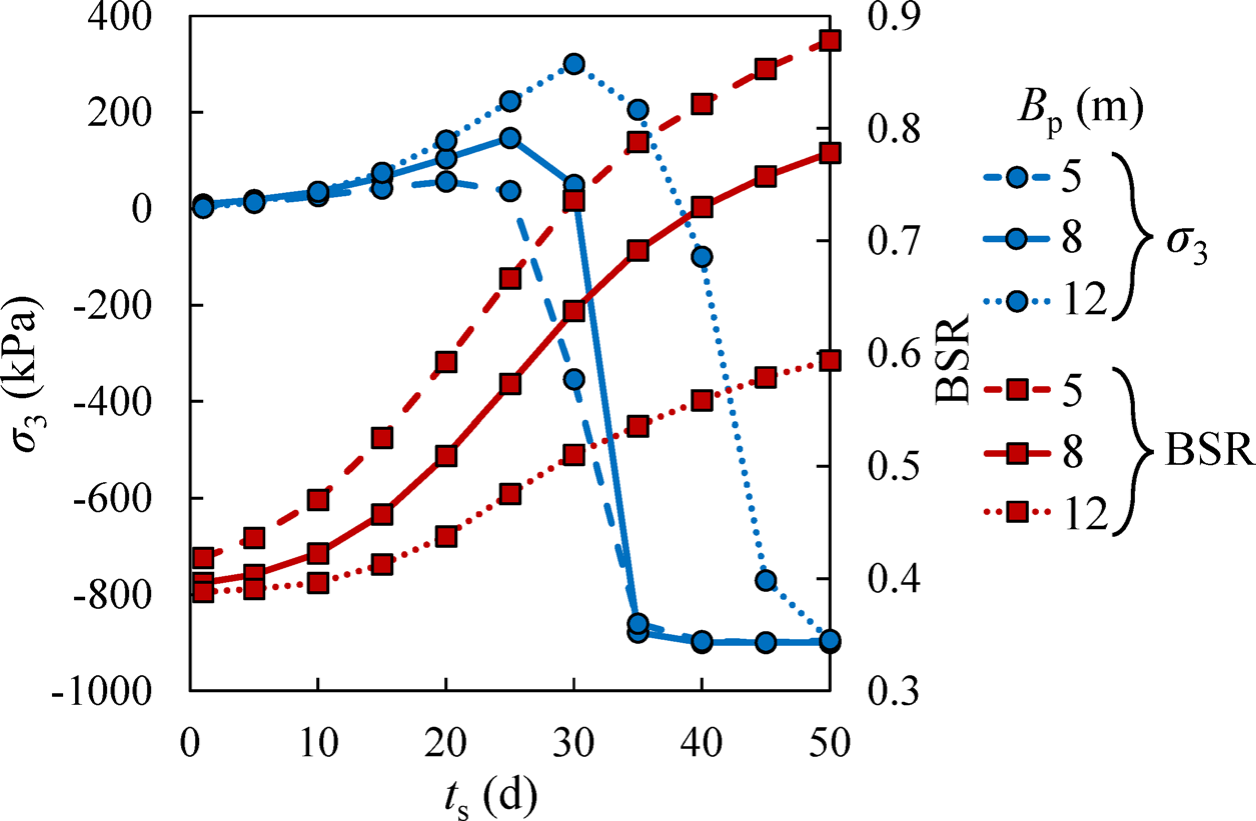
Variations of *σ*_3_ at the point *M* and BSR at the point *N* as functions of *t*_s_ after the extraction of Stope B for different pillar widths *B*_p_ in Case 4.

Figure 11 depicts the evolutions of *σ*_3_ at the point *M* and BSR at the point *N* as functions of *t*_s_ upon extraction of Stope B for different pillar widths *B*_p_ in Case 4. When *t*_s_ is less than 25 d, the increasing trend of *σ*_3_ in the outer region of the intervening pillar is more prominent as *B*_p_ increases from 5 to 12 m. It is because a narrow pillar demonstrates more deformations upon excavation of Stope B that release the confinement applied by the consolidated backfill in Stope A. Furthermore, the wider pillar exhibits the reduction of *σ*_3_ at a longer *t*_s_ with a slower rate. The BSR in the inner part for the pillar also apparently reduces with the increase in *B*_p_. This is explained as a wider pillar is more capable to sustain the swelling pressure applied by highly consolidated backfill during a long *t*_s_, without demonstrating significantly lateral bending effect. The narrow pillar in sequential stope-and-fill operations within squeezing rock condition is thus more critical to not only the sloughing due to tensile condition, but also the brittle damage resulted from the swelling pressure by consolidated backfill.

### Mine depth

**Fig. 12:**
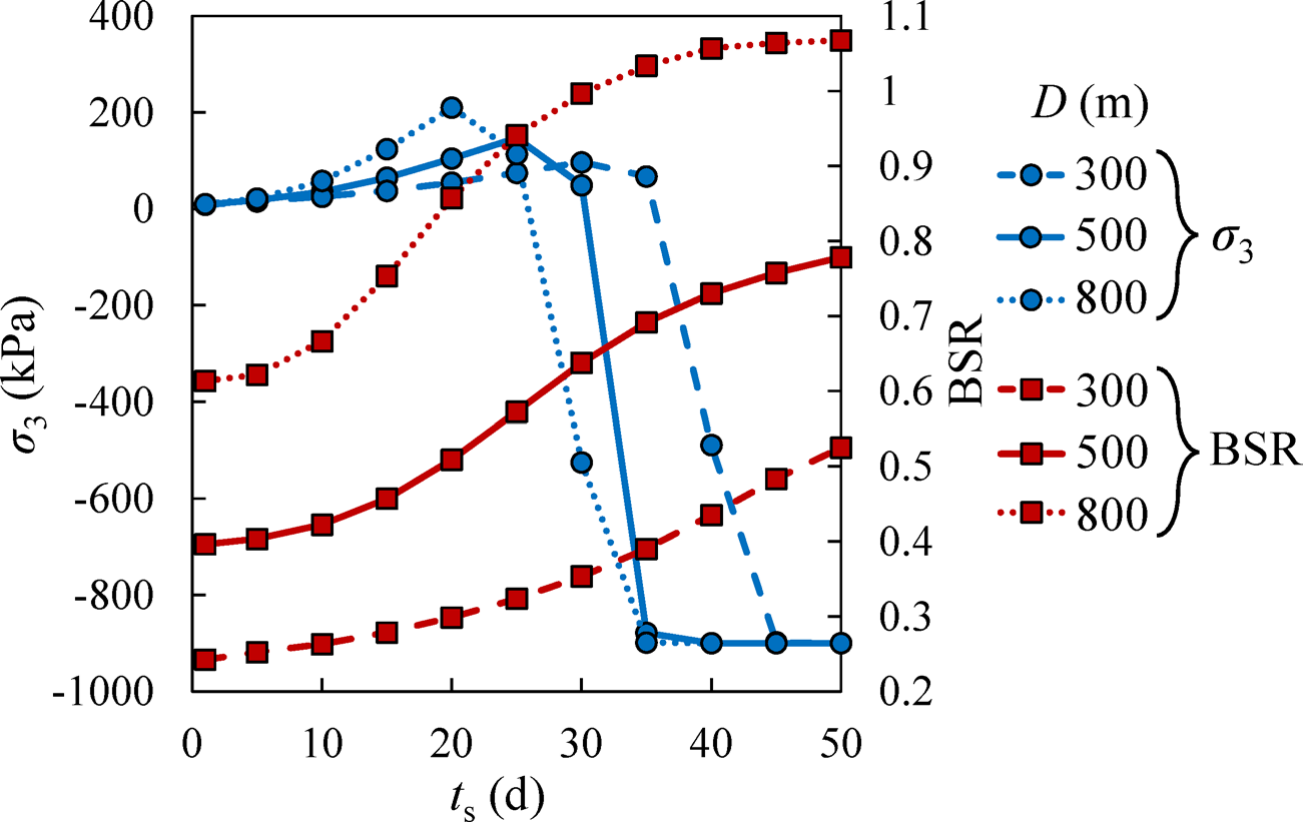
Variations of *σ*_3_ at the point *M* and BSR at the point *N* as functions of *t*_s_ after the extraction of Stope B for different mine depths *D* in Case 5.

Figure 12 illustrates the variations of *σ*_3_ at the point *M* and BSR at the point *N* as functions of *t*_s_ immediately after excavating Stope B for varied mine depths *D* in Case 5. The in-situ stress state elevates with the increase in *D* that leads to the escalation of rock walls creep deformation and larger degree of consolidation of backfill in Stope A. Thereby, as *D* increases, the bending effect of intervening pillar during the extraction of Stope B intensifies, reflected by the reduction of *σ*_3_ at an earlier *t*_s_ and the significant growth of BSR as shown in Fig. 12. Nevertheless, one should note that increasing *D* from 300 to 800 m enhances confinement effectiveness in the intervening pillar with a larger *σ*_3_ when *t*_s_ < 20 d, resulted from greater swelling pressure of consolidated backfill at a larger mine depth.

### Compressibility of backfill

**Fig. 13:**
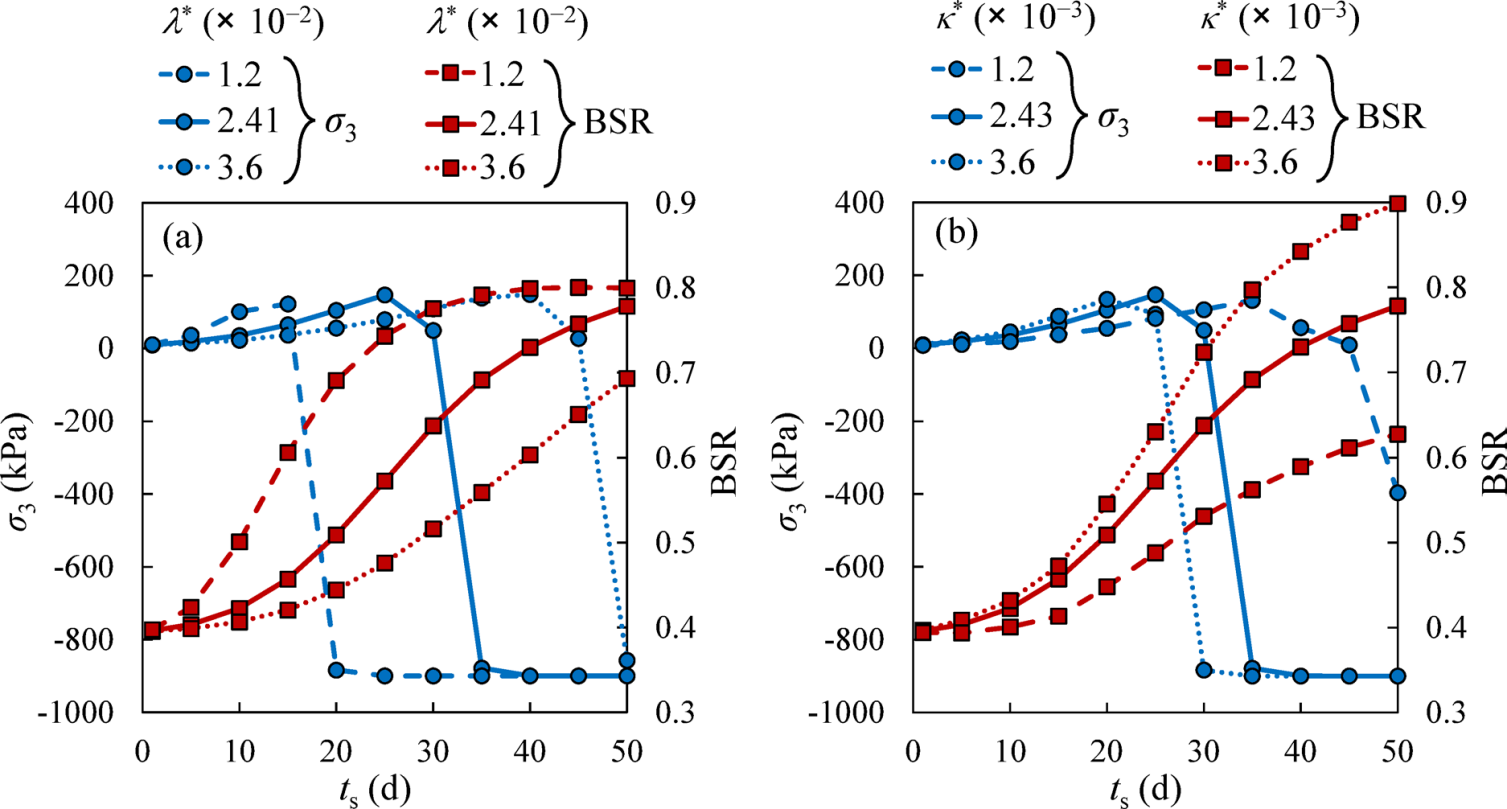
Variations of *σ*_3_ at the point *M* and BSR at the point *N* as functions of *t*_s_ after the extraction of Stope B for varied modified compression indexes λ^*^ and the modified swelling indexes κ^*^ in Cases 6 and 7.

The compressibility of backfill is quantified by its modified compression index λ^*^ and the modified swelling index κ^*^^41–43,53^. Figure 13 draws the variations of *σ*_3_ at the point *M* and BSR at the point *N* as functions of *t*_s_ after the extraction of Stope B for varied λ^*^ and κ^*^ in Cases 6 and 7. As λ^*^ decreases from 3.6 × 10^− 2^ to 1.2 × 10^− 2^, the bending effect of intervening pillar becomes more pronounced as shown by the larger increasing rate of *σ*_3_ in compression condition, the occurrence of tensile condition with shorter *t*_s_ and the increased BSR magnitude. It is because that a smaller λ^*^ corresponds to the lower compressibility of backfill which absorbs larger compressive stresses during rock walls creep deformation, leading to more significant bending of pillar upon excavating Stope B. Meanwhile, Fig. 13b shows that with the increasing κ^*^, the increasing rate of confinement with *t*_s_ elevates around the outer part of pillar, but the tension (negative *σ*_3_) appears earlier with shorter *t*_s_. The BSR in the inner region of the pillar demonstrates an increasing tendency as κ^*^ increases. This is explained as the increased κ^*^ causes larger volumetric expansion (swelling deformation) of backfill in Stope A upon extraction of Stope B. It further results in the intensified bending effect in pillar during adjacent extraction. Therefore, the geomechanical performance of pillar during sequential stope-and-fill operations is affected by the volumetric hardening behavior of backfill. When the stope sequencing time is relatively small, the confinement effectiveness in the pillar increases with the reduced λ^*^ and the increased κ^*^. However, as *t*_s_ further increases the stability of pillar deteriorates with the decrease in λ^*^ and the increase in κ^*^.

### Rheological behavior of rocks

**Fig. 14:**
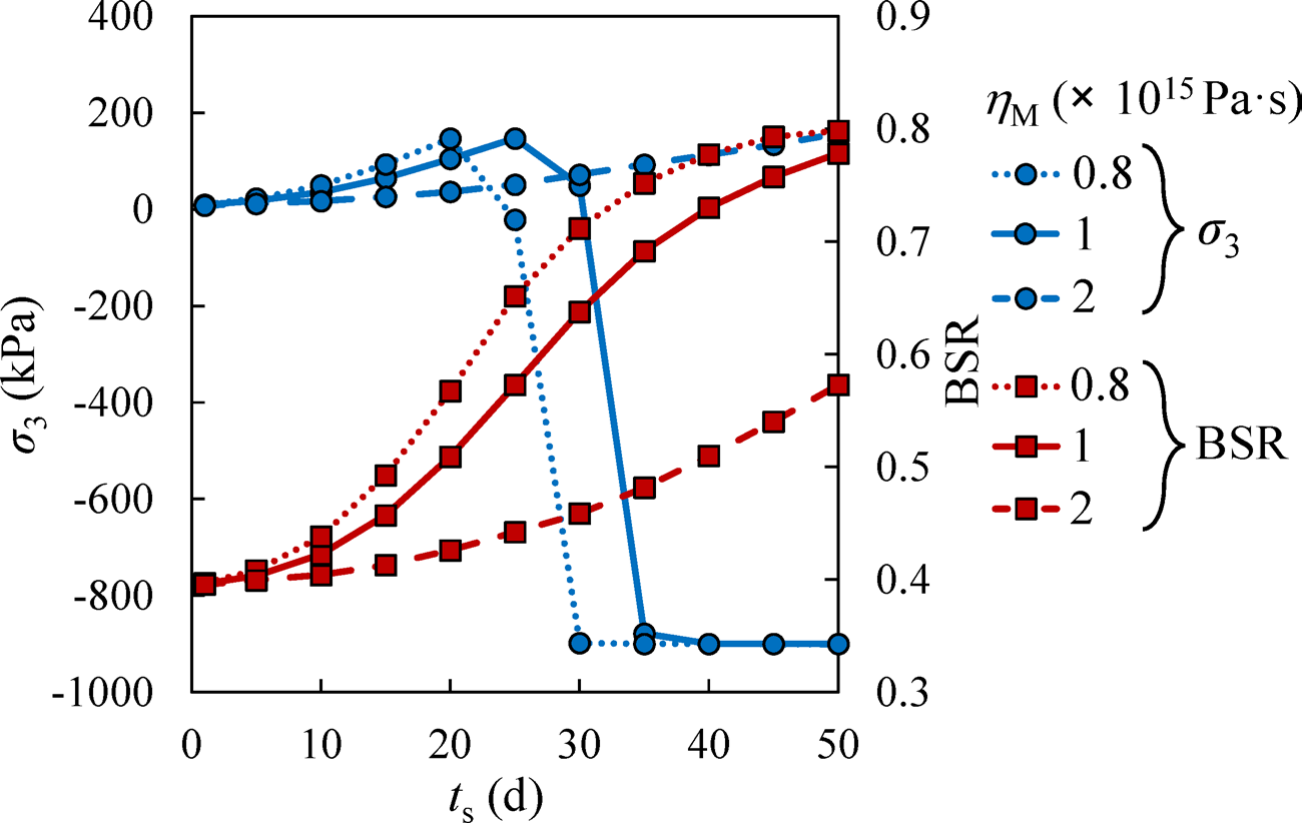
Variations of *σ*_3_ at the point *M* and BSR at the point *N* as functions of *t*_s_ after the extraction of Stope B for varied Maxwell viscosity coefficients *η*_M_ in Case 8.

Maxwell viscosity coefficient *η*_M_ dominates the time-dependent deformation of surrounding rocks^59,60,62^. Figure 14 shows evolutions of *σ*_3_ at the point *M* and BSR at the point *N* as functions of *t*_s_ upon extraction of Stope B for different *η*_M_ in Case 8. In the figure, one sees that the confinement effectiveness of pillar indicated by *σ*_3_ in compression improves as *η*_M_ reduces. Meanwhile, as *η*_M_ increases from 0.8 × 10^15^ to 1 × 10^15^ Pa∙s, the tensile condition caused by bending response in the outer part of the pillar appears with longer stope sequencing time *t*_s_, whereas within the *t*_s_ range in this study, *σ*_3_ in the outer region monotonically elevates once *η*_M_ further increases to 2 × 10^15^ Pa∙s. Moreover, the BSR in the inner portion of the pillar decreases with the increases in *η*_M_. It is explained as the surrounding rocks with a larger *η*_M_ demonstrate less creep deformation, resulted in lowly consolidated backfill in Stope A and less swelling pressure upon extraction of Stope B. Therefore, in squeezing rocks with low *η*_M_, the intervening pillar in sequential stope-and-fill operations experiences more pronounced lateral confinement under short mining cycle durations, but its overall stability significantly deteriorates when *t*_s_ is large.

## Discussion

The geomechanical performance of an intervening pillar during sequential stope-and-fill operations were, for the first time, investigated based on a plane strain numerical model incorporating the creep deformation of rocks and consolidation behavior of backfill. The effect of stope sequencing interval time on the mechanical behavior of pillar immediately upon extracting and after backfilling the second stope was analyzed. Results reveal a bending behavior of pillar with movement towards the newly extracted stope under swelling pressure by the consolidated backfill in the first stope, which further leads to a pronounced asymmetry in the stress state of pillar and affects its stability. Backfilling the second stope can improve the stability of intervening pillar. More parametric analyses were conducted to illustrate the influence of stope geometry, pillar width, mine depth, compressibility of backfill and rheological behavior of rock on the results. This study helps understanding of typically how the sequential stope-and-fill operations and mining cycle interval can affect the geomechanical performance and instability risk of intervening pillar. Through appropriately adapting for site-specific conditions, the developed model can be combined with engineering criteria used for underground design to examine the instability potential for various mining cycle time and sequence alternatives, thus optimizing the mining strategy and maximizing the mine benefits from the ore reserve. Nevertheless, some important simplifications and limitations of this study merit discussions.

The numerical model simplifies actual sequential stope-and-fill mining conditions. In practice, stopes and pillars often have irregular, inclined geometries, whereas the model assumes vertical rectangular shapes. Stope excavation was simulated in a single step, approximating sublevel or long-hole stoping while methods like vertical retreat mining involve staged exposure of the pillar, which may alter pillar response. The model also omits nearby excavations, blasting-induced vibrations, and geological complexities such as discontinuities that cause heterogeneity or anisotropy in pillars. Addressing these factors will be important in future studies to better capture time-dependent pillar geomechanical responses in sequential stope-and-fill mining.

The objective of this study is to reveal the general time-dependent geomechanical responses of intervening pillar in sequential stope-and-fill operations instead of analyzing a specific project. For a given project, measured in-situ stresses along varied orientations can be incorporated in numerical model by applying boundary pressures or initializing stresses throughout the domain. Geological factors such as groundwater, discontinuities, and rock quality can be accounted for using Rock Mass Rating (RMR) system developed by Bieniawski^77^, which provides estimates of shear strength parameters and deformation modulus. The viscosity coefficient can be calibrated against field-measured closure data from underground openings. However, further research is needed to establish a quantitative relationship between RMR and rock mass viscosity.

In this study, an initial lateral earth pressure coefficient of 1 was used to establish the initial equilibrium state, consistent with time-dependent modeling practice^5,45,56,57^. A different initial coefficient would introduce differential stresses that triggers creep even before any excavation. However, once equilibrium is reached in a time-dependent model, the stress state generally evolves toward near isotropy regardless of the initial assumption. Nonetheless, in the case of elasto-plastic models, higher initial horizontal stress increases immediate wall deformation, leading to greater backfill confinement that can affect the pillar performance as reported by Wang et al.^18^.

The plane strain numerical model employed in this study implies that the stope and pillar have a very long length along the third dimension. It may overlook the three-dimensional boundary effects in specific cases where actual geometry lacks adequate length along that direction. Further works are needed to develop a three-dimension numerical model considering the effects of third dimension on the geomechanical responses of the pillar.

The creep behavior of rocks was represented with the Burgers-Mohr model, which is in good agreement with creep strain test results of varied rocks^5,43,60,62,78^. Nonetheless, the Burgers‑Mohr model has notable limitations that warrant discussions. It assumes a linear relationship between creep strain rate and the differential stress, whereas laboratory tests exhibit nonlinear time‑dependent behavior, particularly at high stress levels^31,79,80^. The Burgers-Mohr model dose not account for the influence of the confining pressure on the creep behavior^28,30,80^, which may lead to overestimation of pillar deformation in deep mining conditions where high mean stresses prevail. The Burgers-Mohr model is also restricted to the primary and secondary creep and cannot capture the tertiary creep or time-dependent failure. Therefore, more efforts are required to develop more representative creep model and implement within a numerical code. Moreover, the long-term compressibility of backfill under dry condition was considered in this study, whereas the drainage and dissipation of pore water pressure in the backfilled stope were not accounted. The effect of cementation of backfill on its primary stiffness^40,41,81^ was also not considered in this study which should be addressed in future work.

The applicabilities of the creep model for rocks and the Soft Soil model for backfill have been validated in previous works^5,43^ against results of creep test in compression, one-dimensional consolidation tests, consolidated drained triaxial compression tests and in-situ measurements. Nevertheless, more experimental results and in-situ monitoring are needed to fully validate the time-dependent geomechanical responses of intervening pillar in sequential stope-and-fill mining.

## Conclusion

This article presents a novel numerical model for investigating time-dependent geomechanical responses of the intervening pillar during sequential stope-and-fill operations, by explicitly considering the volumetric hardening of consolidated backfill and the long-term creep deformation of squeezing rocks. The results indicate that prior to extraction of the second stope, the backfill in the first stope undergoes consolidation and volumetric hardening with reduced void ratio under rock walls creep deformation, which sustains greater compressive stresses and delivers enhanced passive support on peripheral rocks. However, the most critical condition for the pillar occurs immediately after excavation of the second stope. At this stage, the consolidated backfill exerts significant swelling pressure on the inner side of the pillar (facing the first stope), inducing pronounced beam-like bending and lateral displacement toward the newly opened stope. This leads to a pronounced asymmetry in the stress state of pillar including the increased differential stress in its inner region and the bending affected minimum principal stress *σ*_3_ and lower differential stress in the outer part (adjacent to the second stope) of pillar. The explicit asymmetrical instability risk indicators were determined for the pillar including *σ*_3_ in the outer region and the brittle shear ratio BSR in the inner portion.

The simulations illustrate that a relatively short stope sequencing interval enables the intervening pillar to benefit from lateral confinement upon excavation of the second stope, owing to the swelling pressure exerted by the partially consolidated backfill. This lateral confinement promotes a lower instability risk state and improves by narrower stope spans, larger pillar widths, greater mining depths, increased backfill swelling index, and reduced backfill compressibility and rock viscosity. These findings support practical design such as using wider pillars and limiting stope spans in deep squeezing ground, and underscore the need to keep sequencing intervals short in production schedules to maintain beneficial confinement from backfill swelling.

The results also show that the overall stability of intervening pillar upon adjacent extraction deteriorates as stope sequencing time further increases, which manifests as the tension in the outer region and increased BSR in the inner portion of pillar. The rate and severity of this deterioration are governed by key geometric and material parameters. For narrow primary stopes, the mining cycle time should be explicitly shortened to suppress the spalling potential for the intervening pillar, whereas greater cautions are needed for the brittle shear failure of pillar neighboring large-span stopes. The narrow pillar is more critical to both sloughing due to tensile stress and the brittle damage resulted from the swelling pressure by consolidated backfill. The stability of intervening pillar further deteriorates with lower backfill compression index and reduced viscosity coefficient of rocks, whereas the shallower mine depths and a smaller backfill swelling index mitigate the adverse effects of prolonged stope sequencing intervals on the pillar stability risk. The results imply that backfill mix designs in practice should balance swelling capacity and compressibility, and sequencing plans should be calibrated to local rock mass creep behavior to avoid compromising intervening pillar performance. The results also indicate that after filling the second stope, the stability of pillar generally improves.

## Data Availability

The data used and analyzed during this study are available from the corresponding author upon reasonable request.
